# Malignant renal epithelioid angiomyolipoma associated with abdominopelvic hydatid cysts: a case report

**DOI:** 10.1186/s13256-015-0556-1

**Published:** 2015-04-10

**Authors:** Youssef Mahdi, Kaoutar Znati, Ali Iken, Zakiya Bernoussi, Fouad Zouaidia, Ahmed Jahid, Yassine Nouini, Najat Mahassini

**Affiliations:** 1 Department of Pathology, Ibn Sina University Hospital, Ahmed Balafrej Avenue, 10000 Rabat, Morocco; 2 Department of Urology, Ibn Sina University Hospital, Ahmed Balafrej Avenue, 10000 Rabat, Morocco; 3 Faculty of Medicine and Pharmacy, Mohammed V Souissi University, Mohamed Belarbi El Alaoui Avenue, 6203 Rabat, Morocco

**Keywords:** Epithelioid angiomyolipoma, Hydatid cysts, Immunohistochemistry, Kidney

## Abstract

**Introduction:**

The World Health Organization defines epithelioid angiomyolipoma as a potentially malignant mesenchymal neoplasm characterized by proliferation of predominantly epithelioid cells and as closely related to the triphasic (classic) angiomyolipoma. It can be benign, potentially aggressive or malignant. The pathologist's role is crucial in making a positive diagnosis, providing appropriate patient management and assessing prognosis. In this report, we present a case of a patient with an epithelioid angiomyolipoma and hydatid cyst association. To the best of our knowledge, such an association has not been reported previously in the literature.

**Case presentation:**

A 70-year-old Arabian woman presented to our hospital with a 6-month history of a right lumbago and weight loss. Computed tomography objectified a mid-right renal tumor, several locoregional lymph nodes and four abdominopelvic cystic formations. The patient underwent a right nephroureterectomy and removal of abdominal and pelvic masses. Histologically, the tumor corresponded to a proliferation of large eosinophil cells, polygonal or ovoid, with epithelial appearance, and associated with thickened, hyalinized vessel walls, fat cells and bundles of smooth muscle cells. Mitoses were estimated at 2 per 50 high-power fields. In immunohistochemical study, epithelioid tumor cells expressed S-100 protein and Melan-A. The diagnosis of malignant epithelioid angiomyolipoma was made. The wall of the abdominopelvic cysts was eosinophilic and lamellar, corresponding to the cuticular membrane of hydatid cysts.

**Conclusion:**

In our patient, careful histological examination and immunohistochemical study allowed us to make the correct diagnosis of angiomyolipoma in its malignant form. The association with hydatid cysts is what makes our case original.

## Introduction

The World Health Organization (WHO) defines *renal epithelioid angiomyolipoma* (AML) as a potentially malignant mesenchymal neoplasm characterized by proliferation of predominantly epithelioid cells and closely related to the triphasic (classic) AML [1]. The latter is composed of a mixture of thick-walled blood vessels, smooth muscle cells and adipose tissue [1-3]. Epithelioid AML can be benign, potentially aggressive or malignant [1,4-6]. The diagnosis is histological. Immunohistochemistry has a major interest in the positive and differential diagnosis. The pathologist should also search for aggressive histological criteria, crucial to appropriate patient management and prognosis assessment. In this report, we present a case of a patient with epithelioid angiomyolipoma and hydatid cyst association. To the best of our knowledge, such an association has not been reported previously in the literature.

## Case presentation

**Figure 1:**
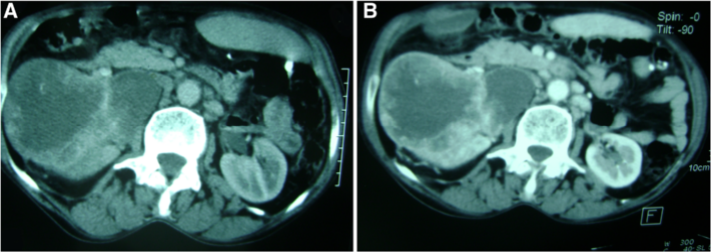
**Tomodensitometric scans reveal a mid-right renal tumor measuring 12cm. (A)** The tumor is breaking the renal capsule. **(B)** Peripheral contrast-enhanced image.

**Figure 2:**
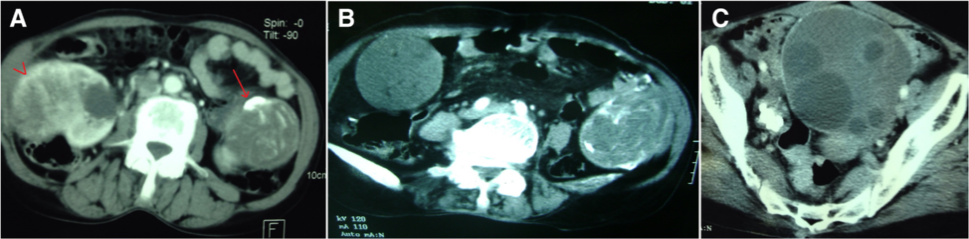
**Tomodensitometric scans show renal tumor as well as abdominal and pelvic cystic formations.** In addition to renal tumor **(A, arrow),** tomodensitometric scans reveal abdominal **(A, arrow; B)** and pelvic **(C)** cystic formations, some of which are calcified.

A 70-year-old Arabian woman presented to our hospital with a 6-month history of a right lumbago and weight loss. She had no personal or family history of tuberous sclerosis. Her physical examination revealed a mass occupying the right side of the lumbar region. Computed tomography (CT) was performed, which demonstrated a large heterogeneous mass measuring 12cm within the middle of the right kidney, with venous thrombosis and several lymph nodes of the renal pedicle and interaortocaval regions (Figure 1). The left kidney had no anomaly. It also showed the presence of four abdominopelvic cystic formations measuring between 3.3cm and 10.5cm, some of which were calcified (Figure 2). These radiological findings favored the diagnosis of malignant renal tumor associated with abdominopelvic hydatid cysts. Thus, the patient underwent a right nephroureterectomy and removal of abdominal and pelvic masses. A midline incision from the xiphoid process to the pubic symphysis was made. After detachment of the right colon and ligature of the right renal pedicle, a right nephrectomy was performed. Then, packing of the operative field with povidone-iodine-soaked sponges was used. Finally, dissection and resection of intraperitoneal abdominopelvic cysts were performed.

**Figure 3:**
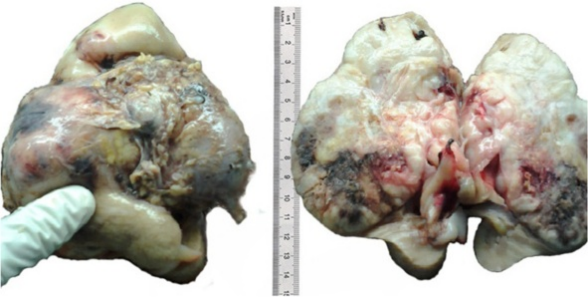
**Macroscopic appearance of the renal tumor**. The tumor occupied almost the entire kidney. It was whitish, firm and had hemorrhagic and mucoid rearrangement. It infiltrated the entire renal pelvis and renal hilum.

**Figure 4:**
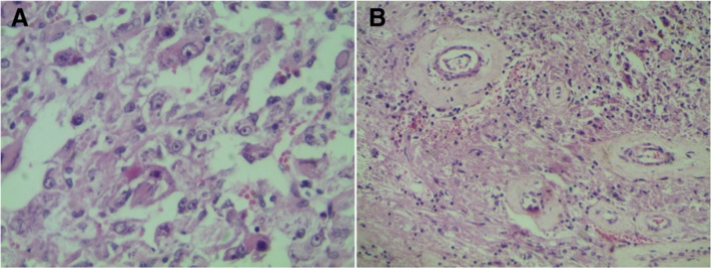
**Representative photomicrograph of the renal tumor. (A)** Epithelioid neoplastic cells were globular, eosinophils, ovoid or polygonal, and had enlarged vesicular nuclei, often with prominent nucleoli (hematoxylin and eosin (H&E) stain; original magnification, ×400). **(B)** Thick-walled and hyalinized blood vessels can be observed in this image (H&E stain; original magnification, ×200).

**Figure 5:**
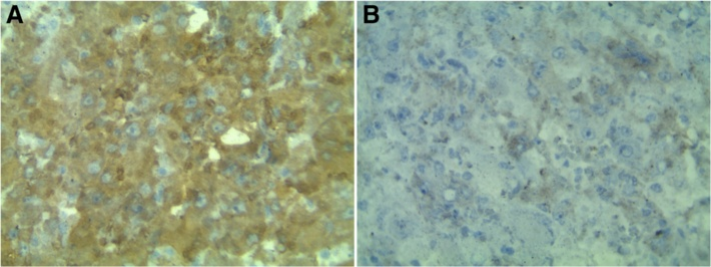
**Immunohistochemical stains of the renal tumor.** Epithelioid neoplastic cells are positive for S-100 protein **(A)** and Melan-A **(B)**.

**Figure 6:**
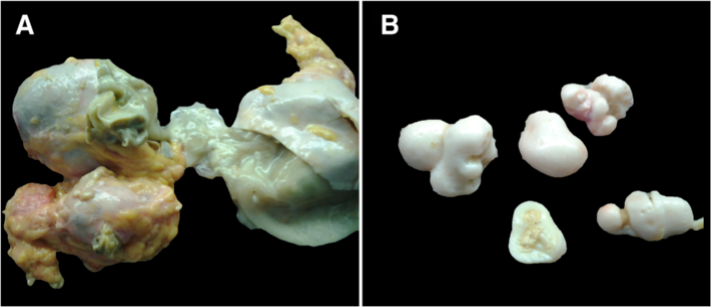
**Gross examination of the cysts. (A)** Gross examination of the abdominopelvic cysts revealed multiple daughter cysts. **(B)** Some of these cysts were calcified.

**Figure 7:**
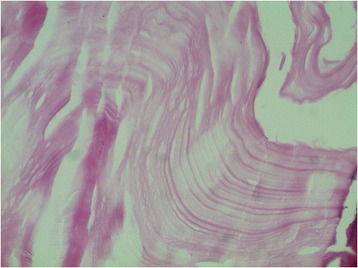
**Histological appearance of the abdominopelvic cysts.** The stain shows an eosinophilic and lamellar membrane corresponding to cuticular membrane of hydatid cysts (hematoxylin and eosin stain; original magnification, ×200).

Gross examination revealed that the renal tumor was whitish and firm and measured 12x11x6 cm. It showed patchy areas of hemorrhage and necrosis. The tumoral capsule was focally ruptured, and the tumor infiltrated the renal hilum and pelvis (Figure 3). Histologically, it corresponded to a proliferation of large eosinophils cells, polygonal or ovoid, with epithelial appearance, associated with thickened-wall hyalinized vessels, fat cells and bundles of smooth muscle cells (Figure 4). Mitoses were estimated at 2 per 50 high-power fields. There was no nuclear anaplasia, vascular invasion or infiltration of perirenal fat. One lymph node was found at the renal hilum and was reactive. In immunohistochemical study, epithelioid tumor cells expressed melanocytic markers: S-100 protein and Melan-A (Figure 5). However, they were negative for anti-pancytokeratin. The diagnosis of malignant epithelioid AML was made. Gross examination of the abdominopelvic cysts showed hydatid membranes. Some of these cysts were calcified (Figure 6). Their wall was eosinophilic and lamellar, corresponding to the cuticular membrane of hydatid cysts (Figure 7). Thoracoabdominopelvic CT showed no distant metastasis of the renal tumor. The patient was lost to follow-up.

## Discussion

In this report, we describe a case of a patient with epithelioid AML and hydatid cysts in a 70-year-old woman. This association was most likely incidental.

Renal epithelioid AML was recognized as a new entity in the 2004 WHO classification of renal tumors. The first cases were described in 1997 [7]. Additional cases were described in the following year [2,3,8]. This is a rare tumor, affecting less than 1 patient per 10,000 population [5]. The number of cases reported in the literature is relatively small. A Cleveland Clinic group reported 16 epithelioid AMLs by reviewing 209 AMLs surgically treated during a 26-year period [9]. The age of presentation ranged from 25 to 47 years, with a mean of 42 years and with a female predominance (80%) [9]. There are two clinical forms of epithelioid AML—sporadic and syndromic in association with tuberous sclerosis (TS) [4,5,9-11]. Our patient had the sporadic form. This tumor arises from the perivascular epithelial cell (PEC) of the kidney [2-5,10]. The PEC gives rise to AML by multiplying and differentiating into smooth muscle cells, adipocytes and dystrophic vessels [5]. Some AMLs are characterized by loss of the short arm of chromosome 16 and TS complex 2 (*TSC2*) gene [1,4,5,10]. *TSC2* and *TSC1* are tumor suppressor genes encoding tuberin and hamartin, respectively, and are involved in the pathogenesis of TS [12]. Tuberin and hamartin form an intracellular complex exerting GTPase-activating activity toward Ras homologue enriched in brain (Rheb) protein [12]. Inhibition of Rheb results in inhibition of the mammalian target of rapamycin (mTOR) pathway [12]. Some AMLs have an oncogenic mechanism by loss of heterozygosity of the *TSC2* gene with activation of the mTOR pathway [5]. Some AMLs dedifferentiate into epithelioid AML by loss of p53 [1,5]. This explains why more than half of patients with epithelioid AML have a history of TS [1].

Clinically, patients with sporadic forms of AML are more often symptomatic than those with syndromic forms [9]. Some cases are discovered during TS follow-up [1]. At CT, we distinguish epithelioid AML as low-fat and isodense to paraspinal muscles [1,5,9] from classical epithelioid AML, which is isodense to fat [5]. If there is a suspicion of malignancy, or in the context of TS, thoracoabdominopelvic CT is the gold standard for staging [5].

Macroscopically, the tumor is usually >6cm size, compact, grayish white, and poorly demarcated with hemorrhagic alterations [1,4,10]. In most cases, no classical AML areas are observed [4,6,10]. The diagnosis is histological, made on the basis of nephrectomy or, more rarely, on biopsy. The pathologist's role is twofold: Make diagnosis and search for aggressive histological criteria.

Histologically, there is a proliferation of round to polygonal epithelioid cells with abundant and eosinophilic granular cytoplasm, enlarged vesicular nuclei and often prominent nucleoli [1,4,6-11]. Multinucleated cells may be present [1,4,7,8,11], as well as spindle and clear cells [1,4,6,7,10,11]. Aggressive histological criteria are nuclear anaplasia, mitotic activity, vascular invasion, necrosis and infiltration of perinephric fat [1,5]. Usually, no area of classic AML is observed [4,10,11]. In immunohistochemical study, tumor cells express melanocytic markers (HMB-45, Melan-A) [1-3,5-7,10] and variably smooth muscle markers (smooth muscle actin, muscle-specific actin) [1-4,6,7] and hormone receptors (estrogen and progesterone receptors) [4,6]. An important fact is that they do not express cytokeratin [4,6,7,10].

Epithelioid AML is a benign tumor in two-thirds of cases and aggressive or malignant in the remainder [1,4-6]. Multiple benign sporadic form locations may be renal or extrarenal [5]. Potentially aggressive forms are defined by the absence of locoregional invasion or distant metastases and are characterized by an alteration of the general state or rapid evolution, aspect of indeterminate renal mass on tomodensitometry, a tumor >5cm and aggressive histological criteria [5]. Malignant forms are those with a locoregional extension or distant metastases [5]: hepatic, pulmonary, osseous, neurological, splenic, peritoneal or testicular [1,4-6,10,11].

In our patient, the tumor was epithelioid AML in its malignant form in view of its size (12 cm), renal hilum and pelvis infiltration, presence of lymph nodes of the renal pedicle and interaortocaval regions and mitotic activity.

The main differential diagnostic consideration is a renal cell carcinoma (RCC). It may arise in radiological stage in cases of poor fat epithelioid AML. Conversely, small amounts of fat can also be found within the borders of RCCs that invade renal sinus fat and liposarcomas [9]. On histological examination, epithelioid cells and absence of classic AML areas can make diagnosis difficult. In a retrospective study of five tumors previously reported as carcinoma, three tumors exhibited a phenotype compatible with epithelioid AML [2]. RCC may be associated with epithelioid AML [9]. In all these cases, immunohistochemistry allows the clinician to clarify the diagnosis by showing epithelial marker negativity and melanocytic marker positivity.

The management of renal epithelioid AML is based on radiographic size and associated symptoms [9]. Active surveillance can be performed for benign tumors not exceeding 4cm [5]. To prevent complications, partial nephrectomy is indicated if AMLs exceed 4cm [5]. In the setting of acute hemorrhage, arterial embolization is the treatment of choice [5,9]. For potentially aggressive forms, nephrectomy is indicated [5]. Kidney removal should be total if tumors are >4cm and partial if not [5]. Radical nephrectomy should be indicated for malignant epithelioid AML with locoregional invasion or distant metastases [5]. Adjuvant treatment with doxorubicin appears to be effective in malignant forms with distant metastases and should be discussed in the case of locoregional invasion [5].

## Conclusions

In our patient, radiological exploration objectified renal tumor with locoregional invasion, but it did not lead us to suspect epithelioid AML under any circumstances. Careful histological examination and immunohistochemical study allowed us to make the correct diagnosis of AML in its malignant form. The association with hydatid cysts is what makes our case original.

## Consent

Written informed consent was obtained from the patient for publication of this case report and accompanying images. A copy of the written consent is available for review by the Editor-in-Chief of this journal.

## Abbreviations

AML: Angiomyolipoma
CT: Computed tomography
mTOR: Mammalian target of rapamycin
PEC: Perivascular epithelial cell
RCC: Renal cell carcinoma
Rheb: Ras homologue enriched in brain
TS: Tuberous sclerosis
TSC: Tuberous sclerosis complex
WHO: World Health Organization
